# Gallium-68 labelled RGD PET/CT imaging of endothelial activation in COVID-19 patients

**DOI:** 10.1038/s41598-023-37390-9

**Published:** 2023-07-17

**Authors:** Evelien A. J. van Genugten, Theresa J. van Lith, Frederik M. A. van den Heuvel, Josee L. van Steenis, Romy M. ten Heggeler, Monique Brink, Laura Rodwell, Frederick J. A. Meijer, Daphne Lobeek, Wanda Hagmolen of ten Have, Frank L. van de Veerdonk, Mihai G. Netea, Mathias Prokop, Robin Nijveldt, Anil M. Tuladhar, Erik H. J. G. Aarntzen

**Affiliations:** 1 Department of Medical Imaging, Radboud University Medical Center, Geert Grooteplein Zuid 10, 6525GA Nijmegen, The Netherlands; 2 Department of Neurology, Donders Center for Medical Neurosciences, Radboud University Medical Center, Nijmegen, The Netherlands; 3 Department of Cardiology, Radboud University Medical Center, Nijmegen, The Netherlands; 4 Faculty of Science and Technology, University of Twente, Enschede, The Netherlands; 5 Department of Health Evidence, Section Biostatistics, Radboud University Medical Center, Nijmegen, The Netherlands; 6 Department of Respiratory Diseases, Radboud University Medical Center, Nijmegen, The Netherlands; 7 Department of Internal Medicine, Radboud University Medical Center, Nijmegen, The Netherlands; 8 Department of Immunology and Metabolism, Life and Medical Sciences Institute, University of Bonn, Bonn, Germany

**Keywords:** Medical imaging, Brain imaging, Radiography, Tomography, Cardiology, Neurology, Immunopathogenesis, Infection, Inflammation, Medical research, Infectious diseases, Viral infection

## Abstract

In coronavirus disease 2019 (COVID-19), endothelial cells play a central role and an inadequate response is associated with vascular complications. PET imaging with gallium-68 labelled RGD-peptide (^68^Ga-RGD) targets α_v_β_3_ integrin expression which allows quantification of endothelial activation. In this single-center, prospective observational study, we included ten hospitalized patients with COVID-19 between October 2020 and January 2021. Patients underwent ^68^Ga-RGD PET/CT followed by iodine mapping of lung parenchyma. CT-based segmentation of lung parenchyma, carotid arteries and myocardium was used to quantify tracer uptake by calculating standardized uptake values (SUV). Five non-COVID-19 patients were used as reference. The study population was 68.5 (IQR 52.0–74.5) years old, with median oxygen need of 3 l/min (IQR 0.9–4.0). ^68^Ga-RGD uptake quantified as SUV ± SD was increased in lungs (0.99 ± 0.32 vs. 0.45 ± 0.18, *p* < 0.01) and myocardium (3.44 ± 1.59 vs. 0.65 ± 0.22, *p* < 0.01) of COVID-19 patients compared to reference but not in the carotid arteries. Iodine maps showed local variations in parenchymal perfusion but no correlation with SUV. In conclusion, using ^68^Ga-RGD PET/CT in COVID-19 patients admitted with respiratory symptoms, we demonstrated increased endothelial activation in the lung parenchyma and myocardium. Our findings indicate the involvement of increased and localized endothelial cell activation in the cardiopulmonary system in COVID-19 patients.

*Trail registration*: NCT04596943.

## Introduction

Coronavirus disease 2019 (COVID-19), caused by Severe Acute Respiratory Syndrome Coronavirus-2 (SARS-CoV-2), starts in the respiratory tract followed by a varying course and severity of disease, ranging from asymptomatic to multiple organ failure^1–3^. The distribution of the angiotensin converting enzyme 2 (ACE2) receptor plays a crucial role in the course of the disease because it is known that the receptor is a target for cellular entry for the coronavirus^4–6^. The ACE2 receptor is expressed on pneumocytes and endothelial cells (ECs) in the lungs, as well as on ECs in the vascular system^4–6^. The presence of viral particles has been demonstrated in multiple organs such as the lungs, brain, and myocardium^7–10^. It has been reported that infection by SARS-CoV-2 can modulate the expression of ACE2 receptor^5,6^. Subsequently, this can result in loss of the inhibitory role of ACE2 on the activation of local acting vasoactive peptides and the initiation of an inflammatory cascade, including the recruitment of immune cells and vascular leakage^5,6,11,12^. Increased endothelial activation also results in a procoagulant state that could lead to arterial and venous thrombi, causing pulmonary embolism (PE) and ischemic stroke^6,13^. These events are frequently observed in COVID-19 patients and are strongly associated with poor outcomes^6,14^. The increased incidence of PE and ischemic stroke in COVID-19 as compared to other viral infections hints towards systemic involvement of ECs, rather than local processes^15–17^. Therefore, endothelial activation and dysfunction might be a critical step in the pathogenesis of COVID-19 and may explain the observed phenomena of a procoagulant state, tissue oedema and ischemic events in multiple organ systems^5,13,14^.

In vivo localization and quantification of endothelial activation in COVID-19 patients is pivotal in developing novel treatment strategies and optimizing patient management. Various radiolabelled arginine-glycine-aspartate tripeptide (RGD)-based compounds have been developed to quantify the expression of integrins in vivo^18–20^. Integrins are cell adhesion molecules that are expressed on the surface of endothelial cells and pericytes**.** Gallium-68 labelled RGD (^68^Ga-RGD), binding to integrin α_v_β_3_, was previously examined in head and neck cancer and arterious-venous malformations^21,22^. As increased tracer uptake on PET/CT is correlated to areas with increased endothelial activation, we hypothesized that ^68^Ga-RGD PET/CT would provide further insight into the role of the capillary and larger vessel endothelium in COVID-19.

In this prospective study, we quantified endothelial activation in lung parenchyma, myocardium and carotid arteries in hospitalized COVID-19 patients using ^68^Ga-RGD PET/CT imaging. In addition, Iodine mapping of the lungs with CT subtraction, as a surrogate of pulmonary perfusion, was used to determine whether endothelial activation affects lung parenchyma perfusion^23^.

## Results

### Patients

From October 2020 until January 2021, ten hospitalized patients with COVID-19 were enrolled in this study. Five patients with similar distributions in age and sex, enrolled in a previous trial (EudraCT 2015-000917-31), were used as reference^22^. Baseline characteristics are shown in Table 1. Patients were admitted to the hospital for a minimum of 6 and a maximum of 23 days. The mean (± SD) D-dimer and CRP values on day of acquisition were 1244 (± 821) and 38 (± 41). During the inclusion period the alpha (also B1.1.7) variant was the predominant SARS-CoV-2 variant in the Netherlands.

**Table 1:** Baseline characteristics and pre-existing comorbidities of COVID-19 patients versus reference patients.

	COVID-19 patients	Reference patients
No. of patients in total	10	5
Male, n (%)	7 (70.0)	4 (80.0)
Age, median [IQR]	68.5 [52.0–74.5]	69.0 [64.0–72.5]
BMI, mean (SD)	30.4 (4.5)	25.2 (5.3)
Hypertension, n (%)	2 (20.0)	2 (40.0)
Diabetes Mellitus, n (%)	2 (20.0)	1 (20.0)
Hypercholesterolemia, n (%)	3 (30.0)	3 (60.0)
COPD, n (%)	2 (20.0)	4 (80.0)
Myocardial infarction, n (%)	1 (10.0)	0 (0.0)
Stroke or TIA, n (%)	2 (20.0)	0 (0.0)
Peripheral arterial disease, n (%)	0 (0.0)	1 (20.0)
Coronary Artery Revascularization (PCI/CABG), n (%)	1 (10)	0 (0)
Total hospital stay in days, median (IQR)	8 [6.8–12.0]	–
Time between admission and PET/CT in days, median [IQR]	5 [4.0–6.3]	–
Time between onset of symptoms and PET/CT in days, median [IQR]	15 [11.0–16.3]	–
Oxygen therapy required during hospital stay, n (%)		
Oxygen suppletion therapy: Nasal cannula/non rebreathing mask	9 (90.0)	–
Non-invasive ventilation: Optiflow	1 (10.0)	–
Invasive ventilation: Intubation	0 (0.0)	–
O_2_ need in L/min at start PET/CT, median [IQR]	3 [0.9–4.0]	–
Complications during hospital stay		
Ischemic stroke or myocardial infarction	0 (0.0)	–
Pulmonary embolism	2 (20.0)	–

*BMI* body mass index, *IQR* interquartile range, *COPD* chronic obstructive pulmonary disease, *TIA* transient ischemic attack, *PCI* percutaneous coronary intervention, *CABG* coronary artery bypass grafting.

### ^68^Ga-RGD PET/CT imaging of the lungs

**Figure 1:**
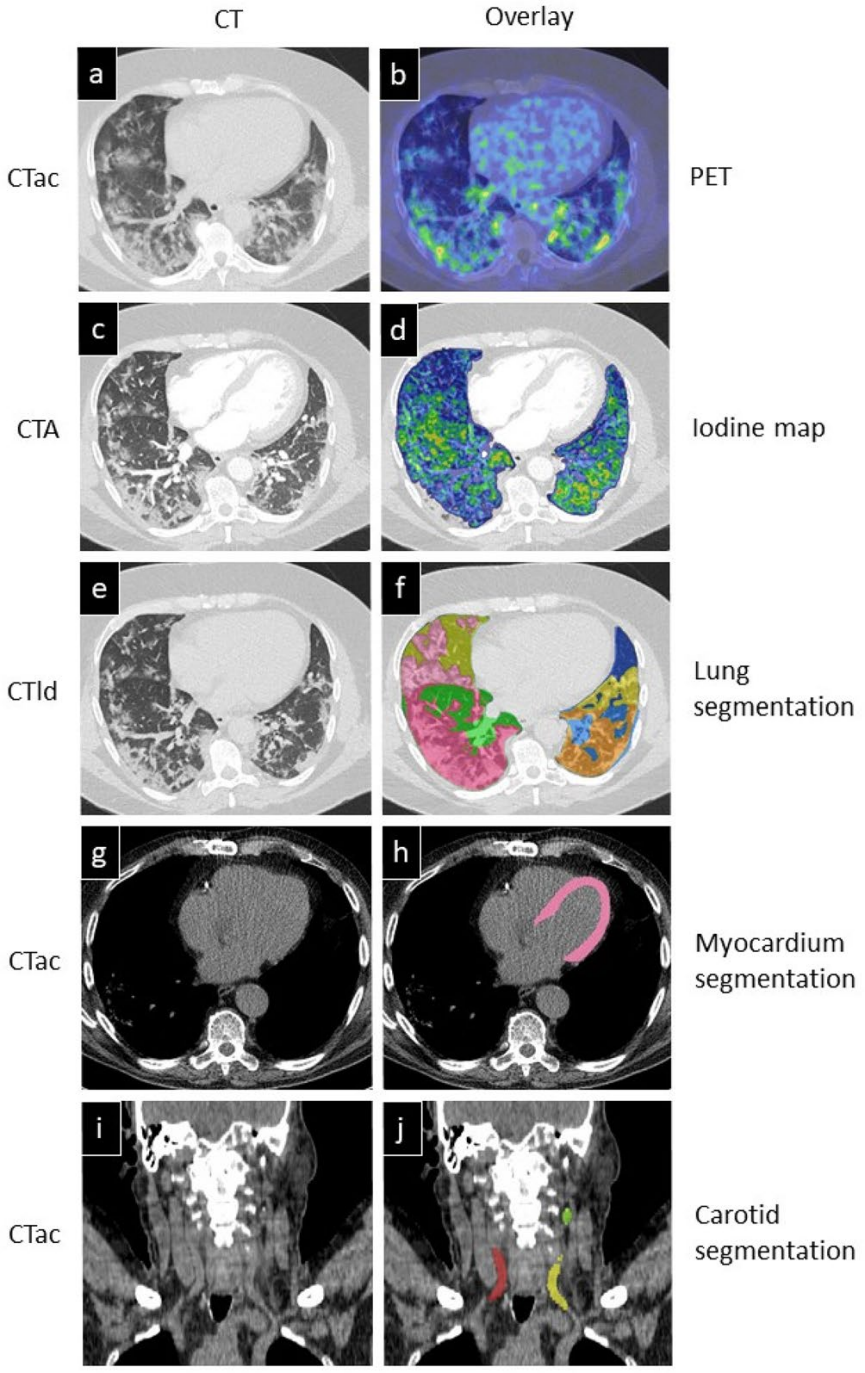
CT reconstructions with correlating PET, subtraction CT iodine mapping and segmentation overlays. (**a**) Axial view of CTac imaging of the thorax. (**b**) CTac imaging of the thorax with attenuation corrected PET overlay. (**c**) CTA of the thorax with breath hold after contrast injection. (**d**) Subtraction CT of the lungs: iodine map overlay. (**e**) CTld of the thorax with breath hold instruction. (**f**) CTld with lung segmentation overlay; Unaffected parts of lobes are colored green and blue. Lilac, pink, orange and yellow are segmented affected parts of the lobes (**g**) CTac imaging of the myocardium. (**h**) CTac with myocardium segmentation overlay. (**i**) Coronal view of CTac imaging of the head and neck. (**j**) CTac with carotid arteries segmentation overlay (red = right carotid artery, yellow = left carotid artery, green = left internal carotid artery).

**Figure 2:**
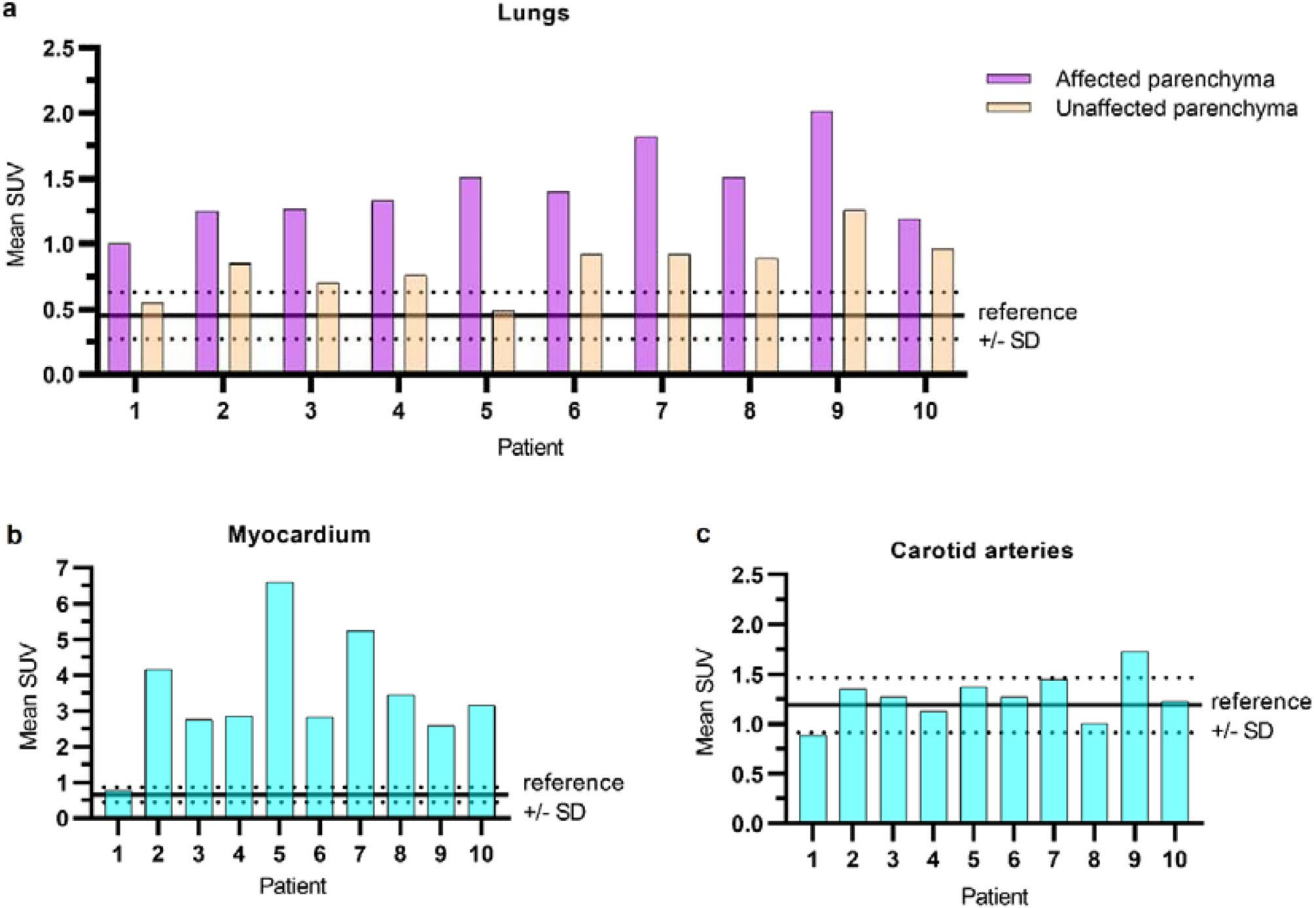
Mean SUV as compared to mean + /− SD (dotted lines) SUV of reference patients in the (**a**) automatically segmented affected and unaffected pulmonary parenchyma per patient, (**b**) myocardium and (**c**) carotid arteries. Abbreviations: SUV : Standardized Uptake Value; SD: Standard Deviation.

To quantify RGD uptake, we calculated the standardized uptake value (SUV) to compensate for differences in net injected activity, incubation time and body weight (CTac, used for attenuation correction, shown in Fig. 1a and PET overlay in Fig. 1f). Mean uptake of ^68^Ga-RGD in the whole lung parenchyma of COVID-19 patients (mean SUV 0.99 ± 0.32) was increased compared to reference patients (mean SUV 0.45 ± 0.18) (*p* < 0.01) (Fig. 2a). A deep learning algorithm for automatic segmentation of parenchymal involvement per lobe was used to define regions of affected lung parenchyma (ground glass opacities (GGOs), consolidations and reticular opacities), and regions without these features (unaffected parenchyma) (Fig. 1h)^24^. Tracer uptake in affected lung parenchyma (mean SUV 1.43 ± 0.30) was increased compared to unaffected parenchyma in all patients (*p* < 0.01). Moreover, mean SUV in unaffected lung parenchyma (0.83 ± 0.22), was also increased compared to reference patients (*p* < 0.01) (Fig. 2a).

**Figure 3:**
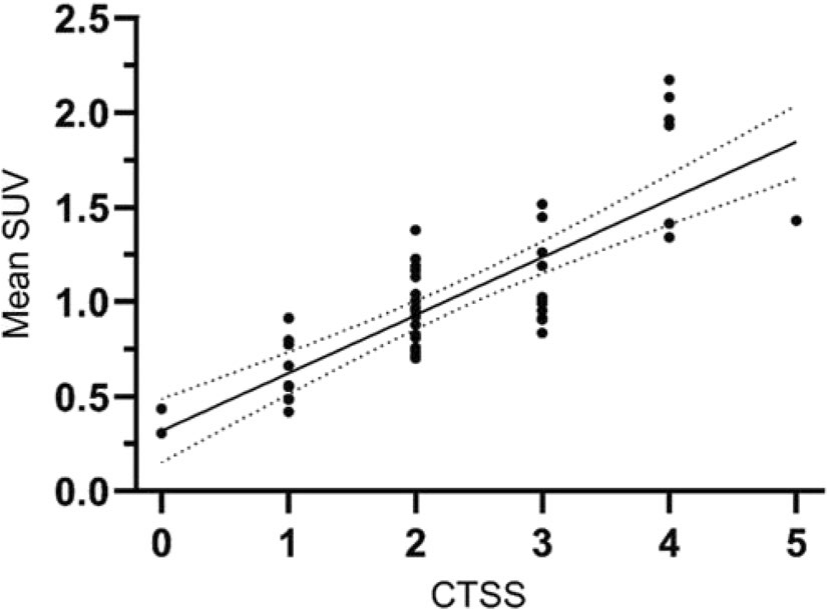
Correlation of CT severity score (CTSS) per lung lobe and mean SUV in COVID-19 patients. Abbreviations: SUV: Standardized Uptake Value. The dotted lines represent 95% confidence interval.

As a measure of severity of involvement of the lung parenchyma in COVID-19 patients, the CT severity score (CTSS) was calculated automatically on basis of the amount of affected parenchyma on the CTld (Fig. 1c)^24^. The CTSS was scored for each lung lobe individually and is found to be correlated (R = 0.80; *p* < 0.01) to the mean SUV of ^68^Ga-RGD uptake per lobe, visualized in Fig. 3.

#### CT subtraction lungs

The association of endothelial activation with perfusion of lung parenchyma was evaluated on a CT subtraction scan (Fig. 1g) with regional differences in distribution of iodinated IV contrast in pulmonary arterial phase (Fig. 1b) used as a marker of pulmonary perfusion. In 2 out of 10 patients the CT subtraction scans were not analyzed due to registration artefacts and an incomplete acquisition of the lungs. Consequently, in 8 patients, 40 lobes were first scored based on their image quality. 0% of lobes were regarded poor, 58% acceptable and 42% good. The main reasons to downgrade diagnostic acceptability were contrast-related beam hardening and scattering around the mediastinum and subclavian artery. Additionally, some parenchymal areas with severe consolidations were not included in the automated delineation of lung parenchyma by the subtraction algorithm and were subsequently excluded from analyses.

**Figure 4:**
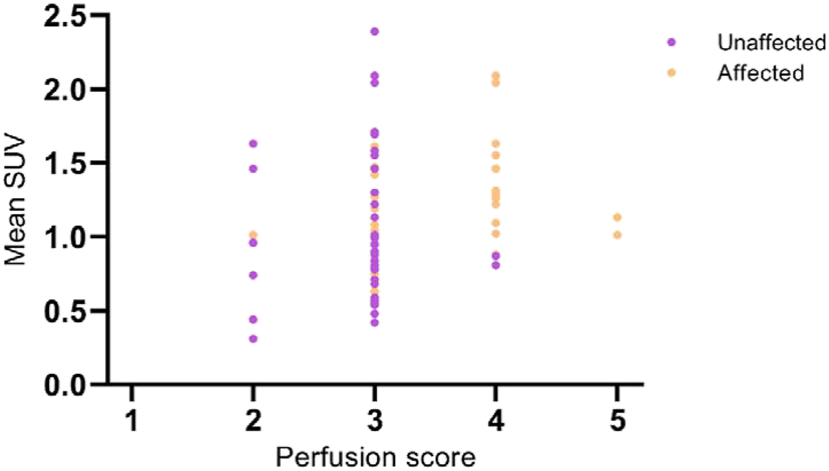
Mean SUV compared to visually assessed perfusion score of automatically segmented affected and unaffected regions per lung lobe. Abbreviations: SUV: Standardized Uptake Value.

Visual scores on a scale of 1–5 of presence and grade of perfusion inhomogeneities in the resulting 37 segmented regions of affected and unaffected parenchyma are presented in Fig. 4. Three out of 40 regions of affected parenchyma were too small to score. Increased perfusion was more often observed in affected lung parenchyma (16/37 regions) as compared to unaffected regions (2/40 regions).

### ^68^Ga-RGD PET/CT imaging of the myocardium

Dyspnea in COVID-19 may not only result from pulmonary causes, but also from cardiogenic causes. Therefore, endothelial activation in the myocardium of the left ventricle was quantified. The mean SUV of the myocardium in the COVID-19 patients was significantly increased in all COVID-19 patients (3.44 ± 1.59) as compared to reference patients (0.65 ± 0.22) (*p* < 0.01) (Fig. 2b). We found no significant difference between the mean SUV derived from the AI-based algorithm (3.44 ± 1.59) compared to the manual delineation (2.70 ± 1.04) of the COVID-19 patients (*p* = 0.09). Figure 1i shows an example of the myocardium segmentation using the CTac scans (Fig. 1d).

### ^68^Ga-RGD PET/CT imaging of the carotid arteries

To study whether endothelial activation was confined to the capillaries, or also larger vessels suppling the brain, the mean SUV of the carotid arteries was calculated and compared with the reference group (Fig. 2c). There was no difference in mean SUV in carotid arteries between COVID-19 patients (1.27 ± 0.23) and reference patients (1.19 ± 0.27) (*p* = 0.39). Figure 1j shows an example of the carotid segmentation using the CTac scans (Fig. 1e). Furthermore, mean SUV values of the left (1.25 ± 0.27) and right (1.29 ± 0.27) carotid artery were compared between patients and did not differ (*p* = 0.94).

### ^68^Ga-RGD tracer distribution

To investigate if there were differences in tracer distribution between COVID-19 patients and reference patients, mean SUV in the blood pool (aorta), muscle and spleen were calculated. The mean SUV in the blood pool was similar between COVID-19 patients and references (*p* = 0.1), as well as uptake in the spleen (*p* = 0.9) and muscle tissue (*p* = 0.6).

### ^68^Ga-RGD uptake compared to clinical parameters

**Figure 5:**
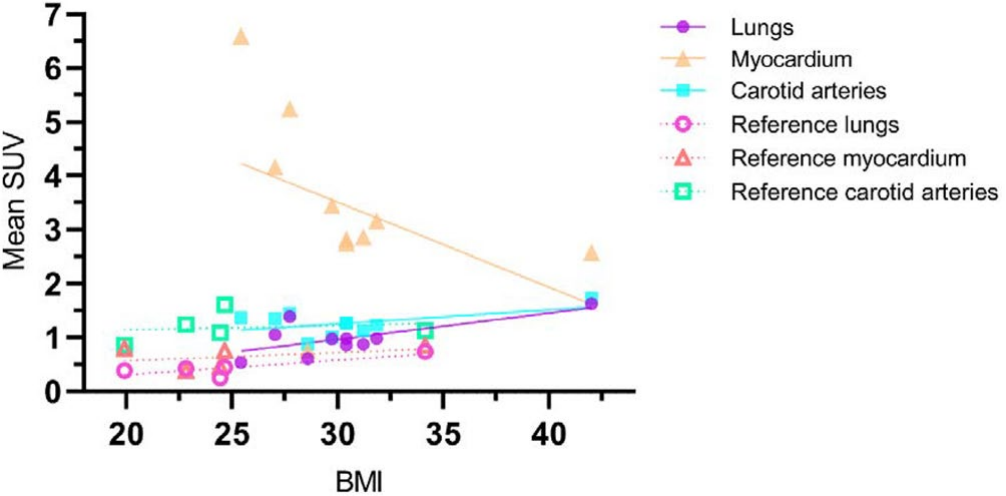
Pearson’s R correlation between BMI and the mean SUV calculated in the lungs, myocardium and carotid arteries per COVID-19 and reference patient. Abbreviations: BMI: Body mass index; SUV: Standardized Uptake Value.

The severity of COVID-19 can be assessed by a variety of clinical parameters, including laboratory markers, medical history and demographic factors (e.g. increased BMI). (5) We investigated if there was a correlation between tracer uptake and clinical parameters associated with severe COVID-19 disease. BMI was significantly positively correlated with mean SUV (RGD uptake) of the lungs of COVID-19 patients (r = 0.68; *p* = 0.03), in the reference group this correlation was not significant (r = 0.81; *p* = 0.10) (Fig. 5). However, a negative correlation between BMI and mean SUV of the myocardium (r = − 0.45; *p* = 0.20) was found in COVID-19 patients (see additional information in supplementary Table S2). The biomarker C-reactive protein as well as the total hospital stay in days of the COVID-19 patients were not significantly associated with tracer uptake in the lungs, carotid arteries or myocardium. For D-dimer, the positive association with mean SUV in the myocardium was significant (r = 0.80; *p* ≤ 0.01) contradictory to the association with lungs or carotid arteries.

## Discussion

In this imaging study, we quantified endothelial activation of lung parenchyma, myocardium and the carotid arteries using ^68^Ga-RGD PET/CT imaging in hospitalized COVID-19 patients with respiratory symptoms. ^68^Ga-RGD uptake was significantly increased in lung parenchyma and myocardium in patients with COVID-19 compared to reference patients. This observation is consistent with endothelial activation in the cardiopulmonary system. Furthermore, endothelial activation was also observed in lung parenchyma that was unaffected on CT images, suggesting a larger involvement of the pulmonary vasculature than is assessed by anatomical imaging by CT. In contrast, no increased uptake in larger peripheral vessels, i.e. carotid arteries, or other organs systems was observed. Therefore, our results suggest specific endothelial activation in the cardiopulmonary system in COVID-19 patients with respiratory symptoms.

COVID-19 induces systemic inflammation^25^ including endothelial activation^5,26^ as part of the physiological response to infection^27^. However, endothelial dysfunction and hypercoagulability are associated with COVID-19 severity^5^ and progression to organ failure^28^. Dysregulation and activation of the endothelium can contribute to the pathological response and may cause collateral damage. Our results confirm the presence of endothelial activation, based on the increased uptake of the tracer in both affected and unaffected lung parenchyma as well as in the myocardium. Because COVID-19 predominantly involves the respiratory tract, these effects in the affected lung parenchyma were expected. Interestingly, we found that in unaffected lung parenchyma (parenchyma without abnormalities on CT) of COVID-19 patients there was still a significantly higher uptake of the tracer compared to lung parenchyma of the reference group. This suggests that endothelial activation is part of the inflammatory response and very likely precedes structural changes in lung tissue in these regions^29^. This may lead to an underestimation of lung involvement in COVID-19 at the time of CT-scanning.

Furthermore, our data indicates that endothelial activation, as part of the inflammatory response, occurs in capillaries throughout the cardiopulmonary system, concluding from significantly increased uptake in the left ventricle. The activation of vascular endothelial cells is also suggested by Nägele et al.^30^ This observed endothelial activation may be one of the reasons for the increased incidence of myocardial infarction, arrythmia and myocarditis during hospitalization and after recovery^31,32^. Moreover, dysfunction of the endothelium due to viral illnesses is associated with long term risk of cardiovascular events^33^. In light of these observations, it is very interesting to notice that we did not observe increased tracer uptake in the carotid arteries. The systemic inflammatory response does not activate the endothelial bed throughout the whole body, but seems to be localized to the cardiopulmonary system.

During the pandemic a high rate of ischemic strokes was reported in hospitalized COVID-19 patients compared to influenza patients (1.5% vs. 0.2%)^34^. Although tracer uptake was not different between patients and references in our cohort, this result does not necessarily imply absence or presence of endothelial activation. For example, the partial volume effect which will affect measurements in small ROIs, has a larger effect for ^68^Ga-labeled tracers than in previously reported studies with ^18^F-labeled RGD-peptides^35^. The resulting underestimation of tracer accumulation hampers a conclusive observation of carotid artery activation in COVID-19 patients.

Generally, obesity is related to endothelial dysfunction^36^, and large retrospective cohort studies previously identified high BMI as risk factor for poor outcome in hospitalized COVID-19 patients^37^. In our limited dataset, in subjects with higher BMI we observed higher tracer uptake in lungs and carotid arteries, but discrepantly lower uptake in the myocardium.

Chest CT was frequently used during the COVID-19 pandemic for diagnosing and risk stratification methods^24,38,39^ Ventilation/perfusion single photon emission tomography (V/Q SPECT) is an alternative to CT angiography, which is able to assess cumulative, real lung perfusion. Several small studies using V/Q SPECT demonstrated heterogenous perfusion patterns in affected lung parenchyma, occasionally colocalizing with COVID-19 related abnormalities on CT^40–43^. In line with these series, our results support the notion that local perfusion defects found in molecular imaging can precede structural changes on CT. Alternatively, the one phase CT subtraction CT protocol with fluctuating image quality might not have been sensitive enough to detect all lung perfusion abnormalities.

There are however several limitations for our study. The study has a relatively small sample size and reference cohort, due to challenging logistics of a molecular imaging study with a short-lived radiotracer in hospitalized COVID-19 patients. Also, the segmentation algorithm used for discriminating affected from unaffected parenchyma may have been influenced by pre-existing pulmonary abnormalities or atelectasis, interstitial lung abnormalities or pulmonary edema, which could not be checked as no previous scans were available. This may have led to a larger area of parenchyma classified to be “affected’’ and therefore possibly lower SUV in the calculation of mean SUV of the affected areas. It is currently unknown if COPD in the reference patients may lead to a higher SUV due to endothelial activation or a lower SUV due to emphysema in the reference measurements. If any, this might underestimate the effect of activated endothelium in affected segments in COVID-19 patients.

In conclusion, we demonstrate that ^68^Ga-RGD PET/CT imaging allows to assess the localization and magnitude of endothelial activation in the cardiopulmonary system in hospitalized COVID-19 patients. Our findings support the hypothesis that endothelial activation is a critical step in the inflammatory response to SARS-CoV-2 infection.

## Materials and methods

### Patients

Ethical permission was obtained from the medical ethical committee Arnhem-Nijmegen (ClinicalTrials.gov identifier: NCT04596943). All study proceedings were performed in accordance with Dutch clinical trials guidelines and all participants provided written informed consent prior to participation. In this single-center proof-of-concept prospective observational study, we included hospitalized adult patients with PCR proven SARS-CoV-2 infection, admitted to the nursing ward. Exclusion criteria included previously documented severe lung abnormalities, glomerular filtration rate ≤ 30 ml/min, contra-indications for PET/CT (pregnancy, breast-feeding or severe claustrophobia) or contra-indications for administration of iodine-containing agents. Patient data, including demographics, medical history, clinical parameters, laboratory examinations, treatment and complications during hospital stay were collected. No adverse events were reported.

#### Non-COVID-19 patients

Five patients with oral cavity squamous cell carcinomas were used as reference. ^68^Ga-RGD PET/CT scans were performed according to the protocol described in the 2016 study of Lobeek et al.^22^.

### Image acquisition

#### ^68^Ga-RGD PET/CT

[^68^Ga]Ga-DOTA-E-[c(RGDfK)]_2_ was synthesized at the Radboudumc (Nijmegen, the Netherlands) as described in Lobeek et al.^22^ A mean dose of 196 ± 20 MBq ^68^Ga-RGD was injected intravenously as a bolus over 1 min followed by saline flushing. All PET/CT scans were performed on a Biograph mCT 4-ring clinical scanner without ECG or respiratory gating (Siemens). PET acquisition of patients 2–10 commenced median 31 min (IQR 24–38) post-injection at 5 min per bed position. Patient 1 was scanned at a significantly later time point post-injection (118 min) due to patient transport logistics, this subject showed a remarkably lower ^68^Ga-RGD uptake across all analyses. The scan range included the thorax, head and neck of the patients. Reconstruction of PET images with vendor specific software comprised of attenuation correction with CT and TrueX algorithm with point spread function and time of flight measurement using 3 iterations and 21 subsets (Siemens). Slice thickness was 3 mm, pixel spacing 4.07 mm, matrix size 200 × 200 voxels and pixel full-width half maximum 3 mm. A 3D Gaussian filter kernel of 3 mm was used for postprocessing.

Low-dose CT scans for attenuation correction (CTac) and anatomical reference were acquired with automatically modulated X-ray tube voltage and current (120 kV, 50 mA). Scan range was equal to PET, slice thickness 3 mm, pixel spacing 0.98 mm, matrix size 512 × 512 voxels and images were reconstructed using a B31f kernel.

#### Subtraction CT

Directly following PET/CT, patients underwent subtraction CT on the same scanner: A CT of the thorax before and after iodinated intravenous contrast administration (iomeprol 300mgl/ml), to evaluate one-phase iodine enhancement of the pulmonary parenchyma. An unenhanced CT (CTld, mean DLP 124 mGy.cm) was made after breath hold instruction with automatically modulated X-ray tube current (reference 75mAs, > 66). Subsequently, injection of a bolus (112 ± 12 ml) of 300 mg/ml iodine-contrast at 5 ml/s was followed by a 40 ml saline chaser at the same injection rate. One patient received a contrast bolus of 3 ml/s and consequently a triggering delay. After a threshold of 100 hounsfield units (HU) was measured in the pulmonary trunk, a breath hold instruction was given to the patient for the acquisition of CT angiography (CTA) of the thorax using modulated current (reference 100 mAs, > 86, mean DLP = 169 mGy cm). Both CT images were acquired with tube voltage of 100 kV and reconstructed with kernel I30f/3, a slice thickness of 1.5 mm with pixel spacing of 0.72 mm and matrix voxel size 512 × 512. Median HU in de pulmonary trunk was 414 HU.

From these two scans, iodine maps were calculated by subtracting Ctld from the CTA scans after motion correction and mask segmentation as described in Grob et al.^23^.

### Image analysis

#### Lung segmentation

We used a previously developed COVID-CT artificial intelligence algorithm for segmentation of the five lung lobes in the CTld images^24^. Additionally, this algorithm segmented affected areas (with GGOs and consolidations) from unaffected areas per lobe. This resulted in 10 ROIs per patient, and the corresponding CT severity score per lung lobe as described in Lessmann et al.^24^ The CTSS was not calculated for the reference group, since this score is validated for COVID-19 and not for COPD associated changes in lung parenchyma. Rigid registration of the CTld to the CTac was performed using MevisLab (Fraunhofer Mevis, Bremen, Germany). This transformation was used to register the segmentation to the PET images and subsequently calculate the SUV within the 10 ROIs.

As the PET scan was made during free breathing, one investigator manually adjusted the segmentations to exclude the liver and spleen signal from quantification if their activity concentration was projected over the lower lung.

^68^Ga-RGD PET/CT scans of the reference patients were used as reference PET signal in lung parenchyma unaffected by COVID-19 infection. The CTac images of the references were segmented using the same lung lobe segmentation artificial intelligence algorithm^24^. This segmentation was registered to the PET images and subsequently adjusted to exclude the liver and spleen signal before calculating mean SUV per lobe.

##### Subtraction CT

One investigator (EvG) and one chest radiologist with 6 years of experience in thoracic radiology (MB) evaluated image quality and presence and grade of perfusion inhomogeneities on subtraction CT. They graded image quality of the perfusion maps per lung lobe on a visual grading scale from 1 to 3 (1: bad, 2: acceptable, 3: good). In each of the 10 ROIs per patient perfusion was assessed on a scale from 1 to 5 (1: severely decreased, 2: decreased, 3: as expected, 4: increased, 5: severely increased) compared to what was expected in a corresponding part of healthy parenchyma. In case of discrepancy between the two readers, this was solved in consensus.

#### Myocardium segmentation

The myocardium of the left ventricle (LV) was delineated on the CTld using the artificial intelligence based algorithm “Whole-heart segmentation in non-contrast-enhanced CT” in all patients^44^. On basis of this segmentation, the mean SUV was calculated for the myocardium. Additionally, one investigator manually (FVDH) delineated the LV myocardium on the CTA of the COVID-19 patients. The mean SUV derived from the algorithm was compared per patient with the mean SUV derived from the manual delineation in order to verify the algorithm. The relatively thin wall of the right ventricle was not delineated as SUV quantification would be unreliable on a scan without ECG or respiratory gating.

#### Carotid artery segmentation

One investigator manually (RvL) delineated bilateral carotid vessel structures of the patients and references on co-registered PET/CT slices (Inveon Research Workplace version 4.2, Siemens). She segmented the common carotid artery (extending from the aortic arch until the carotid bulb), internal carotid artery, external carotid artery and the carotid canal and put ROIs in the lumen, vessel wall and atherosclerotic plaques. The carotid bifurcation was excluded from the ROI to prevent influence of the partial volume effect. ROIs were additionally reviewed by a neuroradiologist with 12 years of experience in neuroradiology. The mean SUV was calculated per ROI and for all regions combined.

### Tracer distribution

We set ROIs for blood pool, muscles and spleen to investigate whether variations in tracer distribution between COVID-19 patients and references occurred and calculated SUV mean values. As a representation for blood pool, an ellipsoid (5cm^3^) was drawn in the lumen of the descending aorta. Muscle activity (where uptake is expected to be low) was calculated using an ellipsoid (5cm^3^) in the trapezius muscle and an ellipsoid (10cm^3^) in the spleen.

### Statistical analysis

Patient characteristics are displayed as counts and percentages and median with IQR. The mean standardized uptake value was calculated and the standard deviation. Differences in mean SUV between COVID-19 patients and references were analyzed using the Mann–Whitney U test. A paired T-test was used to calculate differences in affected versus unaffected parenchyma. Correlations between mean SUV and clinical parameters were calculated using Pearson *r.* Two-sided *P* values of less than 0.05 were considered statistically significant. Statistical analysis was performed using SPSS 25 (IBM) and Graphpad Prism 5 software (GraphPad Software).

## Supplementary Information

Supplementary Tables.

## Data Availability

The PET/CT data generated and analysed during the current study are not publicly available due to privacy restrictions but are available from the corresponding author upon reasonable request.

## References

[1] Wu Z, McGoogan JM (2020). Characteristics of and important lessons from the coronavirus disease 2019 (COVID-19) outbreak in China: Summary of a report of 72314 cases from the Chinese center for disease control and prevention. JAMA.

[2] Klok FA (2020). Confirmation of the high cumulative incidence of thrombotic complications in critically ill ICU patients with COVID-19: An updated analysis. Thromb. Res..

[3] Chen N (2020). Epidemiological and clinical characteristics of 99 cases of 2019 novel coronavirus pneumonia in Wuhan, China: A descriptive study. Lancet.

[4] Varga Z (2020). Endothelial cell infection and endotheliitis in COVID-19. Lancet.

[5] Jin Y (2020). Endothelial activation and dysfunction in COVID-19: From basic mechanisms to potential therapeutic approaches. Signal Transduct. Target Ther..

[6] Bonaventura A (2021). Endothelial dysfunction and immunothrombosis as key pathogenic mechanisms in COVID-19. Nat. Rev. Immunol..

[7] Puelles VG (2020). Multiorgan and renal tropism of SARS-CoV-2. N. Engl. J. Med..

[8] Bradley BT (2020). Histopathology and ultrastructural findings of fatal COVID-19 infections in Washington State: A case series. Lancet.

[9] Borczuk AC (2021). Pulmonary pathology of COVID-19: A review of autopsy studies. Curr. Opin. Pulm. Med..

[10] Matschke J (2020). Neuropathology of patients with COVID-19 in Germany: A post-mortem case series. Lancet Neurol..

[11] Teuwen LA, Geldhof V, Pasut A, Carmeliet P (2020). Author correction: COVID-19: The vasculature unleashed. Nat. Rev. Immunol..

[12] van de Veerdonk FL (2020). Kallikrein-kinin blockade in patients with COVID-19 to prevent acute respiratory distress syndrome. Elife.

[13] Bonetti PO, Lerman LO, Lerman A (2003). Endothelial dysfunction: A marker of atherosclerotic risk. Arterioscler. Thromb. Vasc. Biol..

[14] Nachman RL, Rafii S (2008). Platelets, petechiae, and preservation of the vascular wall. N. Engl. J. Med..

[15] Middeldorp S (2020). Incidence of venous thromboembolism in hospitalized patients with COVID-19. J. Thromb. Haemost..

[16] Kaptein FHJ (2021). Incidence of thrombotic complications and overall survival in hospitalized patients with COVID-19 in the second and first wave. Thromb. Res..

[17] Merkler AE (2020). Risk of ischemic stroke in patients with coronavirus disease 2019 (COVID-19) vs patients with influenza. JAMA Neurol..

[18] Lobeek D (2018). In vivo characterization of 4 (68)Ga-labeled multimeric RGD peptides to image alphavbeta3 integrin expression in 2 human tumor xenograft mouse models. J. Nucl. Med..

[19] Kapp TG (2017). A comprehensive evaluation of the activity and selectivity profile of ligands for RGD-binding integrins. Sci. Rep..

[20] Janssen ML (2002). Tumor targeting with radiolabeled alpha(v)beta(3) integrin binding peptides in a nude mouse model. Cancer Res..

[21] Lobeek D (2020). A clinical feasibility study to image angiogenesis in patients with arteriovenous malformations using (68)Ga-RGD PET/CT. J. Nucl. Med..

[22] Lobeek D (2020). Imaging angiogenesis in patients with head and neck squamous cell carcinomas by [(68)Ga]Ga-DOTA-E-[c(RGDfK)]2 PET/CT. Eur. J. Nucl. Med. Mol. Imaging.

[23] Grob D (2019). Imaging of pulmonary perfusion using subtraction CT angiography is feasible in clinical practice. Eur. Radiol..

[24] Lessmann N (2021). Automated assessment of COVID-19 reporting and data system and chest CT severity scores in patients suspected of having COVID-19 using artificial intelligence. Radiology.

[25] Tomerak S (2021). Systemic inflammation in COVID-19 patients may induce various types of venous and arterial thrombosis: A systematic review. Scand. J. Immunol..

[26] Canzano P (2021). Platelet and endothelial activation as potential mechanisms behind the thrombotic complications of COVID-19 patients. JACC Basic Transl. Sci..

[27] Ait-Oufella H, Maury E, Lehoux S, Guidet B, Offenstadt G (2010). The endothelium: Physiological functions and role in microcirculatory failure during severe sepsis. Intensive Care Med..

[28] Ince C (2016). The endothelium in sepsis. Shock.

[29] Jounieaux V, Mahjoub Y, El-Esper I, Rodenstein DO (2021). The importance of lung hyperperfusion patterns in COVID-19-related AVDS. Eur. J. Nucl. Med. Mol. Imaging.

[30] Nagele MP, Haubner B, Tanner FC, Ruschitzka F, Flammer AJ (2020). Endothelial dysfunction in COVID-19: Current findings and therapeutic implications. Atherosclerosis.

[31] Linschoten M (2020). Cardiac complications in patients hospitalised with COVID-19. Eur. Heart J. Acute Cardiovasc. Care.

[32] Xie Y, Xu E, Bowe B, Al-Aly Z (2022). Long-term cardiovascular outcomes of COVID-19. Nat. Med..

[33] Cooke JP, Connor JH, Jain A (2021). Acute and chronic cardiovascular manifestations of COVID-19: Role for endotheliopathy. Methodist. Debakey Cardiovasc. J..

[34] Merkler AE (2020). Risk of ischemic stroke in patients with coronavirus disease 2019 (COVID-19) vs patients with influenza. JAMA Neurol..

[35] Beer AJ (2014). PET/CT imaging of integrin alphavbeta3 expression in human carotid atherosclerosis. JACC Cardiovasc. Imaging.

[36] Kwaifa IK, Bahari H, Yong YK, Noor SM (2020). Endothelial dysfunction in obesity-induced inflammation: Molecular mechanisms and clinical implications. Biomolecules.

[37] Zhou Y, Chi J, Lv W, Wang Y (2021). Obesity and diabetes as high-risk factors for severe coronavirus disease 2019 (Covid-19). Diabetes Metab. Res. Rev..

[38] Afshar-Oromieh A (2021). A comprehensive review of imaging findings in COVID-19—status in early 2021. Eur. J. Nucl. Med. Mol. Imaging.

[39] Prokop M (2020). CO-RADS: A categorical CT assessment scheme for patients suspected of having COVID-19-definition and evaluation. Radiology.

[40] Das JP, Yeh R, Schoder H (2021). Clinical utility of perfusion (Q)-single-photon emission computed tomography (SPECT)/CT for diagnosing pulmonary embolus (PE) in COVID-19 patients with a moderate to high pre-test probability of PE. Eur. J. Nucl. Med. Mol. Imaging.

[41] Cobes N (2020). Ventilation/perfusion SPECT/CT findings in different lung lesions associated with COVID-19: A case series. Eur. J. Nucl. Med. Mol. Imaging.

[42] Bugatti K (2021). alphaV beta6 integrin: An intriguing target for COVID-19 and related diseases. ChemBioChem.

[43] Burger IA, Niemann T, Patriki D, Fontana F, Beer JH (2020). Lung perfusion [(99m)Tc]-MAA SPECT/CT to rule out pulmonary embolism in COVID-19 patients with contraindications for iodine contrast. Eur. J. Nucl. Med. Mol. Imaging.

[44] Bruns S (2020). Deep learning from dual-energy information for whole-heart segmentation in dual-energy and single-energy non-contrast-enhanced cardiac CT. Med. Phys..

