# Supplementary material for: Gallium-68 labelled RGD PET/CT imaging of endothelial activation in COVID-19 patients
Source: Sci Rep. 2023 Jul 17;13:11507. doi: 10.1038/s41598-023-37390-9 (PMC10352333; doi:10.1038/s41598-023-37390-9)
Supplement: Supplementary file 1 — Supplementary Tables. [file 41598_2023_37390_MOESM1_ESM.docx]

**Gallium-68 labelled RGD PET/CT imaging of endothelial activation in COVID-19 patients**

# **Authors**

Evelien A.J. van Genugten^1*^, Theresa J. van Lith^2*^, Frederik M.A. van den Heuvel^3^*, Josee van Steenis^1,4^, Romy M. ten Heggeler^1,4^, Monique Brink^1^, Laura Rodwell^5^, Frederick J.A. Meijer^1^, Daphne Lobeek^1^, Wanda Hagmolen of ten Have^6^, Frank L. van de Veerdonk^7^, Mihai G. Netea^6,8^, Mathias Prokop^1^, Robin Nijveldt^3^, Anil M. Tuladhar^2^, Erik H.J.G. Aarntzen^1*^

**These authors share the first author position*

## Supplemental material

## Tables

**Table S1. Clinical parameters + outcomes per COVID-19 patient. Age and sex are not included due to privacy reasons.**

| Clinical parameters | COVID-19 patients | | | | | | | | | |
| --- | --- | --- | --- | --- | --- | --- | --- | --- | --- | --- |
|  | **1** | **2** | **3** | **4** | **5** | **6** | **7** | **8** | **9** | **10** |
| BMI (kg/m2) | 29 | 27 | 30 | 31 | 25 | 30 | 28 | 30 | 42 | 32 |
| Saturation (%) | 93 | 96 | 95 | 93 | 91 | 94 | 98 | 95 | 93 | 94 |
| O2 need (L/min) | 0.5 | 2 | 4 | 4 | 0 | 3 | 2.5 | 4 | 4.5 | 3 |
| D-dimer (ng/ml) | 640 | 1410 | 1540 | 1360 | 3300 | 500 | 1450 | 790 | 740 | 710 |
| Ferritin (ug/ml) | 2352 | 949 | 1600 | 2491 | 2251 | 720 | 224 | 56 | 1441 | 957 |
| CRP (mg/l) | 39 | 12 | 123 | 9 | 35 | 2 | 49 | 7 | 9 | 95 |
| LDH (U/l) | 257 | 240 | 429 | 323 | 279 | 308 | 369 | 226 | 279 | 348 |
| ALC (U/l) | 1.10 | 1.39 | 0.92 | 2.15 | 0.41 | 3.61 | 1.00 | 1.11 | 2.74 | 0.72 |
| Hospital stay (in days) | 6 | 6 | 9 | 7 | 23 | 12 | 7 | 12 | 10 | 7 |
| Onset symptoms to admission to hospital (time, in days) | 5 | 12 | 10 | 5 | 9 | 13 | 11 | 11 | 9 | 8 |
| Admission to PET/CT (time, in days) | 5 | 4 | 4 | 6 | 4 | 11 | 5 | 6 | 7 | 3 |
| PET/CT to discharge (time, in days) | 1 | 2 | 5 | 1 | 19 | 1 | 2 | 6 | 3 | 4 |
| ICU admission (time, in days) | 1 | 0 | 0 | 0 | 0 | 0 | 1 | 0 | 0 | 0 |
| SUV lungs (mean) | 0.61 | 1.06 | 0.86 | 0.88 | 0.54 | 0.98 | 1.39 | 0.98 | 1.63 | 0.99 |
| SUV myocardium (mean) | 0.77 | 4.16 | 2.75 | 2.86 | 6.60 | 2.82 | 5.25 | 3.45 | 2.58 | 3.16 |
| SUV carotid arteries (mean) | 0.89 | 1.35 | 1.27 | 1.13 | 1.37 | 1.27 | 1.45 | 1.01 | 1.73 | 1.23 |

Abbreviations: BMI: body mass index; CRP: C-reactive protein; LDH: Lactate dehydrogenase; ICU: intensive care unit; SUV: standardized uptake value

**Table S2. Correlation between clinical parameters and SUV mean per organ (using Pearson R two-tailed)**

|  |  | Lungs | | Myocardium | | Carotid arteries | |
| --- | --- | --- | --- | --- | --- | --- | --- |
| Clinical parameters | | **R-value** | **R-value** | **P-value** | **P-value** | **R-value** | **P-value** |
| BMI | | 0.68 | -0.45 | 0.196 | 0.031 | 0.50 | 0.071 |
| CRP | | -0.19 | -0.04 | 0.919 | 0.604 | -0.05 | 0.901 |
| D-dimer | | -0.40 | 0.80 | 0.006 | 0.248 | 0.22 | 0.538 |
| Total hospital stay | | -0.36 | 0.62 | 0.057 | 0.307 | 0.19 | 0.608 |

*Correlation is significant at a p-value of 0.05 (2-tailed)
